# Pharmacokinetics of Chinese medicines: strategies and perspectives

**DOI:** 10.1186/s13020-018-0183-z

**Published:** 2018-05-02

**Authors:** Ru Yan, Ying Yang, Yijia Chen

**Affiliations:** 1 State Key Laboratory of Quality Research in Chinese Medicine, Institute of Chinese Medical Sciences, University of Macau, Taipa, Macao, China; 2 Zhuhai UM Science & Technology Research Institute, Zhuhai, 519080 China

**Keywords:** Chinese medicines, Pharmacokinetic strategy, Pharmacokinetics–pharmacodynamics relevance, Gut microbiota, Global pharmacokinetics–pharmacodynamics

## Abstract

The modernization and internationalization of Chinese medicines (CMs) are hampered by increasing concerns on the safety and the efficacy. Pharmacokinetic (PK) study is indispensable to establish concentration-activity/toxicity relationship and facilitate target identification and new drug discovery from CMs. To cope with tremendous challenges rooted from chemical complexity of CMs, the classic PK strategies have evolved rapidly from PK study focusing on marker/main drug components to PK-PD correlation study adopting metabolomics approaches to characterize associations between disposition of global drug-related components and host metabolic network shifts. However, the majority of PK studies of CMs have adopted the approaches tailored for western medicines and focused on the systemic exposures of drug-related components, most of which were found to be too low to account for the holistic benefits of CMs. With an area under concentration-time curve- or activity-weighted approach, integral PK attempts to understand the PK–PD relevance with the integrated PK profile of multiple co-existing structural analogs (prototyes/metabolites). Cellular PK–PD complements traditional PK–PD when drug targets localize inside the cells, instead of at the surface of cell membrane or extracellular space. Considering the validated clinical benefits of CMs, reverse pharmacology-based reverse PK strategy was proposed to facilitate target identification and new drug discovery. Recently, gut microbiota have demonstrated multifaceted roles in drug efficacy/toxicity. In traditional oral intake, the presystemic interactions of CMs with gut microbiota seem inevitable, which can contribute to the holistic benefits of CMs through biotransforming CMs components, acting as the peripheral target, and regulating host drug disposition. Hence, we propose a global PK–PD approach which includes the presystemic interaction of CMs with gut microbiota and combines omics with physiologically based pharmacokinetic modeling to offer a comprehensive understanding of the PK–PD relationship of CMs. Moreover, validated clinical benefits of CMs and poor translational potential of animal PK data urge more research efforts in human PK study.

## Background

Pharmacokinetics (PK) characterizes drug disposition in the body by studying the time-course of drug concentrations in biofluids and cell/tissue/organ samples and factors governing its absorption, distribution, metabolism and excretion (ADME) processes. PK study is a prerequisite to establish relevance of the activities/clinical benefits to the chemical contents. The information obtained is crucial for lead identification and optimization in drug discovery and dosage regimen design and adjustment in clinical practice. Comparing to the PK study of western drugs which are generally single ingredient with known target, PK characterization of Chinese medicines (CMs) is fraught with tremendous challenges rooted from their chemical complexity (over hundreds ingredients of diverse chemical types in a single constituent herb or a compound formula, wide concentration ranges, distinct physiochemical properties, etc.), undefined targets (multi-target), and unclear mechanisms of actions. These difficulties are further superimposed by interactions with biological systems (different ADME profiles) as well as those among co-existing ingredients. Unraveling PK profiles of CMs requires adopting strategies distinct from that for western medicines, not only coping with the chemical complexity but also treating the CMs and the compound formula as a whole to provide a holistic and mechanistic understanding of the therapeutic benefits of CMs. Recent rapid development in analytical techniques, systems biology, biochemical pharmacology, as well as multivariate data analysis approaches has promoted the evolution of PK strategies to deal with these challenges.

The fascination of CMs lies in the art of constructing a prescription with multiple CMs which act as “monarch”, “minister”, “assistant” and “messenger”, respectively, to enhance efficacy or reduce toxicity in the intended disease therapy. Mechanistic understanding of the compatibility in this ancient combination therapy guided by the traditional Chinese medicine (TCM) principles is another focus and challenge and has been attempted from pharmaceutical, pharmacodynamic (PD) and pharmacokinetic perspectives [1–3]. The PK interactions among constitute herbs of herb pairs or compound formulas were recently reviewed somewhere else [2, 3]. Majority of the work evaluated the toxicity-reducing [4] or efficacy-enhancing [5] effects of combinatorial use through comparing the PK parameters of a few marker compounds or main components of main constitute herbs in the formula with those dosed in the single herb or pure form. Due to the chemical complexity, complex interactions with biological systems as well as the unavailability of authentic compounds and suitable analytical platform in many laboratories, studies on global chemical changes and kinetic shifts are scarce. It was found that absorptive interactions account for two-thirds (32 from 48 reports) of the PK interactions of CMs [2]. This may be ascribed to the oral intake tradition of CMs which makes intestinal absorption the obligatory path for the constituents to reach the blood circulation. P-glycoprotein (P-gp), the major efflux transporter expressed along the intestine, is the major contributor of the absorptive interactions. For example, Schisandra lignans extract is a strong P-gp inhibitor. Single-dose and multi-dose of this extract could increase the plasma exposure (AUC value) of ginsenoside Rb2, Rc and Rd significantly without affecting terminal elimination half-time [6].

PK study is also imperative to predict interactions of CMs with concomitantly dosed western medicines, unravel the PK interactions among co-existing components, validate the different processing methods, as well as guide formulation design. Co-prescription of western medicines and CMs is very common in China. Herbal products are also increasingly incorporated into western health care owing to an increasing awareness of their health-promoting effects and perceived less side effects. Concomitant use of CMs may mimic, magnify, oppose the effect or even cause toxicity of drugs through PD and/or PK mechanisms. Herb-drug interactions (HDI) have received wide attentions in the past decades. For examples, Radix *Puerariae lobatae* (Gegen), not *Salvia miltiorrhiza* radix (Danshen), offsets the anticoagulant effects of warfarin by accelerating cytochrome P450 (CYP)-mediated metabolism of warfarin, increasing activity and expression of vitamin K epoxide reductase while decreasing those of thrombomodulin in rats [7]. Rhein, the major bioactive anthraquinone of many CMs including rhubarb and *Polygonum multiflorum*, could influence the PK and PD of clozapine to alleviate clozapine-induced constipation [8]. Rhein acyl glucuronide, the major metabolite of rhein in human, significantly decreased the transport of methotrexate mediated by human organic anion transporters (hOAT1, hOAT3) in vitro and inhibited excretion and hence increased methotrexate exposure in rats [9]. Non-toxic dosage of ginsenoside Rh2 enhanced the antibacterial effect of ciprofloxacin towards *Staphylococcus aureus* strains through inhibiting NorA-mediated efflux and promoting ciprofloxacin accumulation in the bacteria [10]. Saikosaponin D did not alter the plasma PK of doxorubicin but enhanced the anticancer efficacy by inhibiting tumor growth and P-gp expression [11]. Recent reviews summarized pharmacokinetic HDI studies and offered insights into the mechanisms, consequences, conflicting results and reasons [12, 13]. So far, majority HDI data were obtained from in vitro studies or animal models, requiring extensive efforts to strengthen the translational potential.

The increasing applications of CMs in disease therapy, the tremendous interests in drug discovery from CMs and the growing concerns on the clinical outcome consistency and safety urgently need the development of suitable PK strategies to dissect the multi-component multi-target holistic clinical effects of CMs. This review offers an overview of the evolving PK strategies and provides a perspective on the future PK study of CMs.

## Strategies for PK study of CMs

People believe that, similar to western medicines, CMs also need to meet the following two requirements to elicit effects: significant exposure and suitable retention time in the target organ or tissue. The chemical complexity, unknown targets, combinatorial use tradition guided by esoteric principles (TCM theory), long history of clinical applications of CMs make them distinguishable from western medicines which are usually chemically simple and have definite targets, requesting distinct PK strategies that can establish concentration-activity/toxicity relevance to allow mechanistic insights into the efficacy/toxicity of CMs. However, despite these inherent differences, the majority of previous PK studies of CMs adopted the same strategy tailored for western medicines which usually focus on the systemic exposure (drug levels in blood) of drugs. To cope with the chemical complexity of CMs, major efforts of the PK study of CMs have been laid on selecting representative components as well as improving sensitivity of analytical methods for PK measurement. Thus, considerable research efforts have been devoted to identify or predict in vivo available components of CMs using in silico, in vitro or in vivo approaches and describe their plasma PK profiles [14]. The strategies have evolved from single PK study to PK–PD correlation study, with analytes spanning from quality control chemical marker, major herbal components, selected PK markers, multi-components, to global drug-related components profiling together with host metabolic network shifts adopting metabolomics approaches [15, 16].

### Chemical marker/major component/multi-component PK using classic strategy

The diverse chemical types and the wide concentration ranges of the components in CMs demand excellent analytical capability in both accurate structural identification and sensitive quantitation. Relying on the availability of analytical instruments and standard compounds, earlier PK study of CMs usually investigate the in vivo fates of single components (in pure or mixed form), and gradually assemble the findings into a whole picture. Quality control marker compounds documented in China Pharmacopoeia and/or major components in the herbs were usually chosen for PK studies because the authentic compounds were more easily obtained. They were either dosed as pure compound or in mixed form (extract or fraction) or both to obtain the PK parameters and identify PK interactions with co-existing components. For example, PK of ferulic acid was depicted in normal and blood-deficiency-syndrome rats receiving Fo-Shou-San which is composed of Danggui and Chuanxiong [17]. PK of Z-butylidenephthalide, a bioactive phthalide present in a significantly low quantity in medicinal herb Chuanxiong Rhizoma, was investigated in rats using a Chuanxiong extract, a fraction containing Z-butylidenephthalide and the standard compound, and found that the major compound coexisting in the herb ligustilide can form Z-butylidenephthalide, making the latter one of the major circulating components after oral intake of the herbal extract [18]. However, each CM usually contains hundreds of components of a variety of chemical types which possess diverse physiochemical properties, and as consequences, the PK profile of a single or a few compounds may not describe the PK profiles well or show good relevance to PD measurements of the CMs. Moreover, the chemical markers documented for quality control may not be the abundant or specific in the herb. For example, tetramethylpyrazine and ferulic acid, the two marker compounds used for Chuanxiong Rhizoma and related products, are traceful (< 0.1 µg/g crude drug) [19] and ubiquitously distributed in plant kingdom [20], respectively. Moreover, the major component in the herb may show low systemic exposure due to poor absorption or extensive elimination [21]. The rapid advances in analytical techniques, in particular the LC–MS/MS techniques (Qtrap, QqQ, QTOF, etc.) allow simultaneously identifying and/or monitoring dynamics of multiple components using classic strategy which generally requires prior knowledge of herbal chemistry and is time-consuming [22]. Simultaneously monitoring PK of multiple parent compounds and metabolites (i.e., poly-PK) has only been reported in a handful of studies [23, 24]. For examples, 142 metabolites were identified from bile and plasma samples from rats receiving Danggui Buxue Decoction [25]; more than 60 metabolites were identified and PK profiles of 55 were obtained for metabolites of licorice [26, 27].

### Identification of surrogate PK markers

Simultaneous determination of PK of multiple components in herbal medicines is technically challenging due to the wide concentration ranges, complex interactions with the body/among the co-existing components in ADME processes, as well as diverse elimination dynamics in vivo. Although poly-PK using classic strategy allows simultaneous determination of multi-components, most of the in vivo available components may not show ideal PK properties due to the following reasons: (1) too low systemic exposures in blood to contribute to the efficacy of CMs (PK–PD disconnection), (2) poor dose-exposure relevance (blood exposure does not change proportionally with dose), (3) the metabolites, not the prototypes from CMs, reaching considerable exposure, (4) exposure not relevant to efficacy/safety, (5) unclear targeting tissues/organs/molecules and mechanisms of actions. Moreover, it usually has poor high-throughput (time-consuming), relies on availability of analytical instrument and chemical standards, thus, is not practicable to be applied in other laboratories or readily translated to industry or clinical practice to improve the efficacy, safety, and quality consistency of CMs. In the past decade, Chuan Li’s group has carried out poly-PK studies of many CMs using integrated in vivo–in vitro–in silico approaches [14, 28–30]. The authors advocated the use of surrogate “pharmacokinetic markers” to describe PK profiles of CMs. The surrogate PK markers (prototypes and/or metabolites) of CMs should meet the following requirements at the same time [31]: (1) exhibit significant exposure, (2) show good dose-exposure correlation, (3) exhibit good correlation or prediction of drug efficacy, safety, or factors that affect exposure. For examples, tanshinol from Danshen showed dose-dependent systemic exposure (as judged from the area under concentration–time (AUC) value) and significant correlation between the urinary recovery and its plasma AUC. Oral or sublingual intake of cardiotonic pills which contain Danshen as the major constitute herb showed no differences in absorption and bioavailability of tanshinol. As such, tanshinol was proposed as a promising PK marker for the cardiotonic pills [28]. In rats receiving oral administration of *Panax notoginseng* (Sanqi) extract in rats, ginsenosides Ra3, Rb1 and Rd were identified as PK markers for systemic exposure of the herb due to long-circulating and high exposure levels of the three ginsenosides resulted from their slow biliary excretion, low metabolism, and slow renal excretion [29]. However, in healthy volunteers taking Sanqi extract orally, plasma 20(*S*)-protopanaxadiol (PPD) and 20(*S*)-protopanaxatriol (PPT) were considered as more suitable PK markers which reflect the individual microbial activity, dynamics and inter-individual differences in plasma exposures of respective oxidized metabolites, the major circulating forms of ginsenosides in the blood circulation [30]. Very interestingly, poly-PK study of Danhong injection [Danshen and Carthami Flos (Honghua)] suggested that a combination of the daily dosage with the elimination half-life determines whether a component can serve as an appropriate PK marker to reflect systemic exposure of CM injections [30]. When given alone, berberine showed very low concentration in blood and failed to prevent anaphylaxis reactions in peanut allergic mice, while the intestinal absorption of berberine was significantly enhanced by co-existing components in an herbal formula, leading to remarkable increase of berberine bioavailability and consequent the prevention of peanut anaphylaxis. Thus, berberine was identified as the chemical and PK marker of the compound formula [32].

### Integrated PK of CMs

The chemical components of CMs usually fall into several main different chemical types, each containing tens of compounds bearing a same skeleton with varied substituents/conformations. In vivo metabolism of these structural analogs will produce even more metabolites keeping the same skeleton. Owing to the structural similarity, compounds and their metabolites of the same chemical type possibly exhibit similar biological activities with potency varied to different extents. For each single compound, it may not be detectable or the exposure is too low to allow significant contribution to the clinical outcomes. However, when administered together in a mixture (the CM fraction or extract), these components may produce additive/synergistic effect, contributing significantly to the holistic actions of CMs. Thus, comparing to PK of single compound or individual PK data of multiple effective components, integrated PK property of CMs can offer more comprehensive understanding of the exposure-efficacy/toxicity relevance. Cai’s team detected 191 metabolites of taxifolinb, a ubiquitous bioactive constituent of foods and herbs, in rats receiving 3-day consecutive oral dosing of the compound. These metabolites exhibited a wide distribution in the body and more than 60 metabolites were predicted to have similar targets as the prototype does, suggesting that these metabolites which keep the same pharmacophore as the bioactive parent compound may act on the same targets in vivo and hence produce additive effects [33]. An AUC-weighting integral PK approach was proposed for evaluating the holistic PK characteristics of multiple components bearing the same core structure. Xie et al. found that the integral PK of Schisandra lignans obtained using an AUC-weighting approach correlated well with their hepatoprotective effect and the hepatic injury biomarkers [34]. Considering that different substituents of structural analogs may affect efficacy/toxicity to different extents, Wang and colleagues compared the integrated toxicokinetics of major diosbulbins after oral administration of *Dioscorea bulbifera* rhizome extract using AUC- and IC50-weighting approaches, respectively. The IC50-weighting integrated plasma concentration–time profile showed better correlation with the hepatic injury measurement total bile acids [35], suggesting bioactivity of structural analogs as weighting coefficient offer better integrated kinetics than the exposure data.

### Classic PK–PD study of CMs

Many CMs have well-documented therapeutic benefits and multiple pharmacological activities but elusive targets and mechanisms. The PK profiles of CMs and the PK–PD relationship are key to identify real active components (prototype or metabolite), unravel efficacy/toxicity mechanism of CMs and reveal PK compatibility in a compound formula and predict HDI. An increasing number of studies have included both PK and PD measurements into the efficacy/safety assessment of CMs. Ren et al. found that three chemical types (flavonoids, iridoids, alkaloids) of Huang-Lian-Jie-Du decoction, a compound formula consisting of Coptidis Rhizoma, Scutellaria Radix, Phellodendri Cortex, Gardenia Fruit, and notable for heat-dispersing and detoxifying effects, showed distinct modes of anti-inflammatory activity by determining the concentration-effect relevance between the plasma PK profiles of 41 drug-related components (prototypes and metabolites) and the levels of 7 cytokines in lipid polysaccharides-induced rat inflammation model [36]. A transdermal patch containing glycyrrhetinic acid and paeoniflorin, two primary active compounds in peony-liquorice decoction, exerted a synergistic constant analgesic effect (number of writhes) on dysmenorrhea model mice with a single dose. The pharmacological response versus plasma concentration plot of glycyrrhetinic acid revealed a counterclockwise hysteresis loop [37]. Ginsenoside Rb1 coupled with schisandrin delayed the elimination of ginsenoside Rg1 and the three compounds in a mixture displayed a synergistic effect on NO release [38]. Blood–brain barrier opening property of borneol was well explained by measuring the expression and function of efflux transporters (Mdr1a, Mdr1b and Mrp1) and the distribution of borneol in different brain regions (cortex, hippocampus, hypothalamus and striatum) [39]. These classic PK–PD studies usually focus on one or a few main prototypes/metabolites of the CMs and determined limited biochemical measurements or clinical endpoints which may be not relevant to the biological responses directly elicited at the target organ/tissue. The multi-component multi-target working mode of CMs requires a comprehensive insight into the mechanisms through global analysis of the dynamic changes of CMs and biological responses.

Metabolomics is a technology originally developed to inform what did happen to a biological system (organism, organ, cell, etc.) through comprehensive unbias analysis of small molecules in a biofluid, cell, organ or organism. It is a promising approach to address the challenges in poly-PK and classic PK–PD of CMs when coupled with multivariate statistical tools. Metabolomics can not only decode biological network perturbation to a stimulus by identifying the most significantly affected endogenous metabolites and their metabolic pathways, but also resolve the relationships between endogenous and xenobiotic metabolic processes [40]. Metabolomics has been successfully applied to numerous xenobiotic metabolism studies and to predict drug efficacy and drug-related side effect through the knowledge of metabotype (known as pharmacometabolomics) [41]. Wei Jia and co-workers proposed a poly-PK strategy using metabolomics approach [15], which was recently applied to a study of Huangqi Decoction (consisting of Astragali Radix and Glycyrrhizae Radix) in healthy Chinese volunteers [16]. A total of 56 prototypes of Huangqi Decoction and 292 metabolites were identified and the concentrations of the herbal metabolites were correlated with 166 endogenous metabolites [16], providing an unprecedented level of insight into the mechanism of action for Huangqi Decoction. Undoubtedly, the tremendous analytical capability enables metabolomics a powerful tool in unraveling the mechanisms under the efficacy/toxicity of CMs through analysis of the metabolome to ascertain the perturbations resulting from CMs intervention.

### Cellular PK–PD to address PK–PD disconnection of CMs

Poor plasma concentration-efficacy/toxicity relevance is a common issue for CMs. Most drug-related components (prototype or metabolite) showed poor blood exposure owing to low abundance in the original herb or unsatisfactory in vivo ADME property, thus is believed to be impossible to contribute to efficacy/toxicity of CMs. For instances, ginsenoside Rb1 and Rg1 showed extremely low oral bioavailability due to poor absorption, extensive microbial deglycosylation, biliary excretion, acidic degradation [29, 42]. They showed definite neuroprotective effects, while were hardly detected in brain [43]. The cerebral exposure levels to flavonols and terpene lactones in rats receiving oral administration of GBE50 (a standardized extract of *Ginkgo biloba* leaves) are much lower than the concentrations required to elicit neuroprotective effects in vitro [44]. Although showing a very low systemic exposure (< 10 ng/mL), berberine has demonstrated remarkable anti-diabetic effects in vivo in animals and human which could not be explained by activity observed in vitro at a much higher concentration. To address the PK–PD disconnection of CMs, a cellular PK–PD strategy was proposed which determines the cellular drug accumulation and intracellular drug distribution and correlates the cellular dynamic drug disposition with its intracellular target binding and efficacy [45]. Cellular drug exposure is believed to be more relevant to drug efficacy than plasma drug exposure when drug targets localize inside the cells, instead of at the surface of cell membrane or extracellular space, and hence, cellular PK–PD is complementary to traditional PK in unraveling the action mechanisms of CMs. Cellular PK–PD of some compounds originated from CMs have been summarized in a recent review article [45]. Acidotropic trapping, binding to intracellular sites and carrier-mediated import and export transport systems, contribute to steady-state intracellular of accumulation quinine, an antimalarial component from Cinchona Bark [46]. Comparison of the localization signals of the fluorescent artemisinin derivative with organelle specific dyes revealed that endoplasmic reticulum is the main site of artemisinin accumulation [47]. Anti-oxidation effects of herbal flavonoids kaempferol, galangin correlated to stronger autofluorescence in the nucleus than cytoplasm in hepatocytes visualized by confocal laser scanning fluorescence microscope [48]. In H_2_O_2_ treated neuronal culture, quercetin pretreatment prevented neuronal death from the oxidant exposure although intracellular quercetin or related metabolites were undetectable, suggesting alternative mechanisms of quercetin neuroprotection beyond its long-established ROS scavenging properties [49]. The cellular PK has also been successfully applied to explain the anti-cancer effects of paclitaxel from *Taxus brevifolia* and camptothecin from *Camptotheca acuminate*. Comparing to imaging techniques, in particular fluorescence imaging, cell fraction approach provides an alternative method for drugs having no fluorescence, offering not only intracellular distribution but also accurate drug concentrations [50]. The determinants of drug subcellular distribution include active transport, metabolic inactivation, pH partitioning, electrochemical gradient, and target binding. Among these factors, drug transporters and enzymes are still the key determinants that govern the amount of drugs entering the target intracellular organelle and the corresponding drug efficacy. Particle size is one of the determinants for formulations. Anti-cancer potency and cellular uptake of curcumin micellar nanoparticles are directly correlated to particle size and the smaller nanoparticles are more potent and localized in both nucleus and cytoplasm [51].

### Reverse pharmacokinetics to aid target identification and drug discovery

Acknowledgement of the multifactorial property in the etiology of many chronic diseases has facilitated multi-target drug discovery [52]. A recent review of new molecular entities (NMEs) approved by the US FDA between 2000 and 2015 revealed an increasing number of multi-target NME [53]. Multi-target therapy can be achieved through combinatorial use of existing drugs with known different targets. On the other hand, CMs have shown validated clinical benefits from a long history of use. Many compounds from CMs, such as berberine, curcumin, ginsenosides, and baicalein, have been confirmed to possess diverse pharmacological activities in vivo. Thus, CMs offer an attractive and promising source for discovery of pleiotropic single molecule or multi-component preparations for multi-target therapy. However, the targeting tissues, organs or molecules and mechanisms of CMs are largely unclear. The pleiotropic compounds from CMs generally have low oral bioavailability and could not provide significant exposure and sufficient retention time at the diseased sites which are considered as prerequisites to elicit the pharmacological effects in modern drug discovery. To cope with these challenges in reverse pharmacology guided drug discovery from CMs, a new concept ‘reverse pharmacokinetics’ was comprehensively introduced by Hao et al. [54]. Comparing to conventional drug discovery which evaluate PK desirability of compounds with a definite target to assess their druggability, reverse PK assesses metabolism and PK of CMs and integrate these knowledge with validated clinical benefits/pharmacological activities to aid target identification and mechanistic understanding of the holistic outcomes (efficacy or toxicity), define exposure-efficacy/toxicity relevance, and facilitate discovery of NMEs or multi-target multi-component drugs. Increasing evidence support complex manifestations of many chronic diseases via multiple signaling pathways at remote sites other than directly targeting on the pathological nodes. For example, the neuroprotective effect of ginsenosides could not be well explained by a direct action due to their extremely low brain exposure, rather, it can be attributed to their immunomodulatory and anti-inflammatory activities in the periphery which can interplay with central nervous system and is functionally implicated in the pathogenic development of many brain diseases [43]. Promising evidence suggests that berberine can boost intestinal health partially through balancing gut microbial structure [55], which is in line with its poor plasma exposure, but high exposure and long retention in gut. In contrast, the high hepatic extraction and distribution (70-fold increase in liver) [56] correlates well with the hypolipidemic effect of berberine probably through targeting hepatic low density lipoprotein receptors. Moreover, the reverse PK information can also help design and selection of physiologically relevant in vitro models to evaluate the molecular mechanisms, facilitate efficient drug discovery from CMs, as well as justify personalized medicine in TCM practice.

## Perspectives

In the past decades, numerous PK studies of CMs have been reported owning to a wider recognition of the crucial roles of PK in mechanistic understanding of the multi-component, multi-target holistic benefits of CMs and new drug discovery from CMs. The PK strategies for CMs also evolve faster to meet the growing demands. The ultimate goal is to establish PK–PD relevance of CMs to ensure suitable quality control, pertinent pharmacological evaluation and consistent clinical output, which undoubtedly is crucial but tremendously challenging due to chemical complexity by nature, undefined targets, complex interactions among co-existing compounds and combinatorial use tradition guided by obscure TCM theory, disconnection between disease site and target site, etc. The rapid advances in systems biology, omics, multivariate data analysis approaches allow us to translate the holistic clinical benefits into modern scientific data and bring our understanding of the mystery of the old tradition to unprecedented depths. The future research efforts may consider improving the PK–PD relevance study in the following two aspects.

### Global PK–PD to address presystemic interplay of CMs with gut microbiota

The recent rapid advancement of our knowledge in the physiological, pathological and pharmacological roles of gut microbiota in human also promote an in-depth understanding of its multifaceted roles in drug metabolism, efficacy and toxicity [57] and the holistic therapeutic benefits of CMs [58]. The enormous gut microbial metabolic capability has been well recognized from numerous reports in the past decades, which is demonstrated to be complementary to host drug metabolizing system by generating more permeable metabolites to facilitate intestinal absorption/enterohepatic recirculation, leading to enhanced systemic exposure [59]. Gut microbiota catalyze a variety of reactions of structurally diverse compounds, in particular hydrolysis of glycosides from natural products [60, 61]. The typical example is ginsenosides which undergo stepwise deglycosylation in gut lumen [42] and the more permeable secondary metabolites or aglycones showed higher exposures [14, 29] and were believed to mainly account for the pharmacological activities of ginseng. The chemical complexity and the traditional oral route also favor manipulation of intestinal homeostasis by some ingredients of CMs. An increasing evidence support the beneficial effects of CMs on gut microbiota structure, intestinal inflammation, intestinal epithelial barrier function (P-gp, tight junction, etc.). For instances, Mori Cortex extract can alleviate colitis-like symptoms in dextran sulfate sodium-induced colitis rat model through reinstating microbial balance, regulating inflammatory responses, and up-regulating intestinal P-gp which involved a direct effect and a gut microbiota-mediated mechanisms [22]. It has been well recognized that gut microbiota play a pivotal role in shaping host intestinal immune responses [62]. Recent reports on the crosstalk between gut and other organs, such as the gut- brain, liver, kidney, lung axes [63–65] revealed tight connections between gut microbiota and many diseases, implying gut microbiota as an important potential peripheral target of drug therapy. This may provide another explanation for the disconnection between the therapeutic benefits of CMs in many chronic diseases [66] and undesirable PK profile. The last, scattering data pointed to a third role of gut microbiota in manipulating host drug disposition. Comparative analysis of hepatic gene expression from germ-free and conventionally-raised mice revealed a cluster of 112 differentially expressed target genes predominantly connected to xenobiotic metabolism and pathways inhibiting retinoid X receptor function [67]. A number of gut microbiota derived metabolites, bacterial strains, bacterial components such as outer membrane vesicles, or fecal microbiota transplantation could regulate transporters and drug-metabolizing enzymes or their up-stream regulator nuclear receptors PXR, CAR, PPARs etc. [68–71]. The PK and PD study of calycosin-7-*O*-β-d-glucoside suggested the contributions of gut microbiota to both disposition and efficacy of CMs. We conceived that the holistic health benefits of CMs should be attributed to components that can interact with gut microbiota to manipulate intestinal hemeostasis and those, either prototypes or the metabolites formed by gut microbial metabolism, which can reach the blood circulation to elicit effects [72]. Therefore, it is imperative to include the presystemic interactions with gut microbiota into the PK–PD study of CMs.

**Fig. 1:**
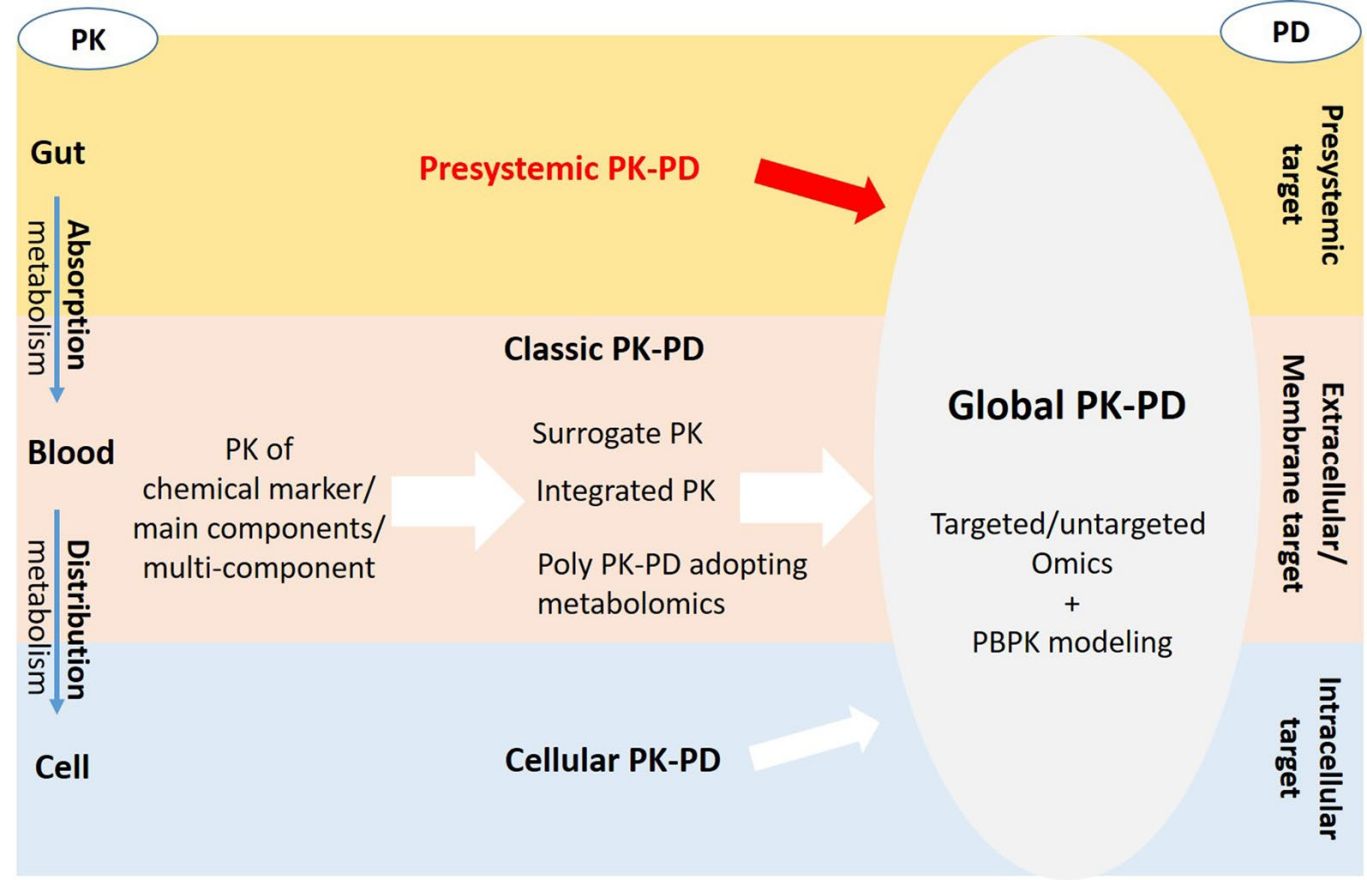
The evolving strategies for the pharmacokinetic study of Chinese medicines. *PK* pharmacokinetics, *PD* pharmacodynamics

Physiologically based pharmacokinetic (PBPK) modeling is a powerful mathematical modeling technique for predicting drug ADME in humans and other animal species through integrating anatomical, physiological, physical, and chemical descriptions [73]. It offers mechanistic insights into the factors determining drug disposition in specifically designated compartment (predefined organs or tissues) and enables personalized medicine by providing precisely characterized individual variability. Including individual gut microbiota information (structure, metabolic activity, etc.) into a physiologically based pharmacokinetic and pharmacodynamic (PBPK/PD) model is a challenging task but will be a promising approach to allow more precise prediction of inter-individual variability in drug disposition and response and assessment of the contributions of gut microbiota to the holistic therapeutic benefits of CMs. Thus, here we propose a global PK/PD strategy which will combine classic PK–PD which measures systemic drug exposure and extracellular and/or membrane targets, cellular PK–PD which examines cellular drug distribution and intracellular targets, with presystemic PK–PD which determines relevance between gut drug exposure and microbial targets, for examples, gut microbiota composition or specific microbial drug-metabolizing activity (Fig. 1). The advantages and disadvantages of classic PK-PD, cellular PK–PD and the newly proposed global PK–PD are summarized in Table 1.

**Table 1:** The advantages and disadvantages of strategies/approaches for the pharmacokinetic study of Chinese medicines

Strategy	Advantages	Disadvantages
Classic PK-PD	PK study adopts the same strategy tailored for western medicines which focuses on systemic exposures PD study usually measures limited pharmacological parameters/clinical endpoints, except for poly PK-PD Can identify bioactive components with ideal PK property for new drug discovery from CMs	PK–PD profiles of limited number of components may not describe the complex dose-exposure-efficacy/toxicity relationship and explain the multi-component multi-target action mode rooted from the chemical complexity of the herb/compound formulas Systemic exposures of most components are too low to account for the holistic benefits of CMs The interactions with gut microbiota prior to intestinal absorption were ignored Components targeting at cellular components show poor PK–PD relevance
* PK of chemical marker/main component/multi*-*component*	Obtains individual PK profile of chemical marker/main components/multi-component of CMs	Restricted by herbal chemistry knowledge, availability of authentic compounds and analytical technology Chemical markers documented for quality control may not be the abundant or specific in the herb Main compounds may not show ideal PK property and be main circulating components
* Surrogate PK*	Describes pharmacokinetic profiles of CMs using surrogate PK markers (prototypes and/or metabolites) which exhibit significant exposure, show good dose-exposure correlation, and exhibit good correlation or prediction of drug efficacy, safety, or factors that affect exposure Less time-consuming, more readily be translated to industry or clinical practice	It’s difficult to find compounds which show both high exposure and good dose-exposure and efficacy correlation The PK profiles of the surrogate marker may subject to changes when the amounts/compositions of co-exiting components vary
* Integrated PK*	Describe the holistic PK characteristics of CMs using the integral PK of components bearing the same core structure Establish dose-exposure and efficacy relationship for a group of, not individual, bioactive components Bioactivity/toxicity-weighting integral PK approach correlated better with efficiency/toxicity	Establishment of structure–activity relationship is limited by the availability of authentic compounds The metabolites of the components which are bioactive and the main circulating form should be included for calculating integral PK Bioactivity/toxicity-weighting integral PK will change with specific bioactivity/toxicity tested
* Poly PK*-*PD*	Applies metabolomics for PK and PD profiling Allows the correlation of the perturbations of endogenous metabolic network with the disposition of the drug-related components Monitors global/specific metabolic shifts using untargeted/targeted metabolomics approaches	The analyte coverage and detection sensitivity rely on the analytical techniques Only those gut microbial metabolites and host-microbial co-metabolites entering the circulating system are possibly detected
Cellular PK-PD	Determines the cellular drug accumulation and intracellular drug distribution and correlates the cellular dynamic drug disposition with its intracellular target binding and efficacy Be more relevant to drug efficacy than plasma drug exposure when drug targets localize inside the cells	Drugs entering cell are limited by transporters and drug-metabolizing enzymes Intracellular drug concentrations are generally low Relies on the specificity and sensitivity of imaging techniques Performed in vitro, complementary to traditional PK to establish PK–PD relevance
Global PK-PD	Combines classic PK–PD which measures systemic drug exposure and extracellular and/or membrane targets, cellular PK–PD which examines cellular drug distribution and intracellular targets, with presystemic PK–PD which determines relevance between gut drug exposure and microbial targets The association study of gut microbial alterations and host metabolic shifts allows estimating the contribution of gut microbiota to the health benefits of CMs Adding a compartment describing individual microbial structure/function data into the PBPK modeling allows more precise prediction of inter-individual variability in drug disposition and response	Requires powerful instrumental platform and multivariate statistical tools to deal with very complex sample analysis and data analysis and interpretation

### Clinical PK–PD study of CMs in patients

So far, majority of the PK knowledge of CMs was obtained from animal models. Advances in molecular biology and pharmacogenetics enable a more comprehensive view of interspecies differences in drug disposition and the underlying physiological and pathophysiological mechanisms. Big differences have been reported between humans and animals commonly used (rat, mouse) for preclinical PK study [74]. Although allometric approaches do allow successful extrapolations of PK data of many western medicines from animals to humans [75], species differences are not only numerous but also sometimes unpredictable, not allowing generalisation. For PK of CMs, the chemical complexity and other factors rooted from it superimpose the species differences, thus preclinical PK data of CMs generally have less translational potential and poorer clinical implications than western medicines.

An increasing number of human PK studies of CMs were reported. Most studied widely prescribed single herbs or famous compound formulas in healthy volunteers at one single oral dose, with single or a few marker/main compounds measured. The impact of inflammation on host drug metabolizing enzymes has been well documented [76, 77]. Changes of drug transporters in diseases accounted for PK alterations of many drugs [78]. Diseases and drug/nutrients interventions cause gut microbiota structure shifts, leading to the microbial metabolic activity changes [79], and as consequences, have impacts on host immune status, drug disposition and efficacy, which will be finally converged to affect the holistic clinical outcomes of CMs. Comparing to the ‘laboratory to clinic’ discovery process of western medicines, CMs have demonstrated to be effective in long history of clinical applications with undefined targets. The ‘clinic to laboratory’ paradigm allows mechanistic insights into the holistic benefits of CMs at clinical relevant dosages with less ethical hurdle in clinical PK study in patients. In a recently released guidance for industry on botanical drug development, the US Food and Drug Administration also requested the sponsor to ‘measure the blood levels of known active constituents or major chemical constituents in a botanical drug product using a sensitive analytical method to achieve the same objectives of Phase 1 and 2 clinical pharmacology studies for non-botanical drugs [80]. Collective efforts from relevant parties (clinic practitioners, pharmacokineticists, pharmacologists, and bioanalysts) are needed for establishing PK–PD relevance to unravel holistic mechanisms under the efficacy/toxicity of CMs in human.

## Conclusion

The chemical complexity undoubtedly is the basis of the multi-target holistic action mode of CMs which makes them attractive, in particular, in an era when more diseases are found to be multifactorial and demand combination drug therapy, while on the other hand, it hampers the mechanistic understanding of their holistic therapeutic benefits. The validated clinical benefits/pharmacological activities, the elusive targets and mechanisms, the undesirable ADME properties and the PK–PD disconnection, appeal for a PK strategy that follows a distinct paradigm from the one tailored for western medicines to address these challenges. The rapid advancement of the analytical techniques, systems biology, and multivariate analysis methods have promoted the development of several PK strategies, allowing the study of PK–PD relevance between the disposition of multiple/global drug-related components and the extracellular/membrane targets and intracellular targets. The emerging enormous evidence support the close connections of gut microbiota with many diseases and its multifaceted role in drug disposition, efficacy, and toxicity. The presystemic interactions of gut microbiota are believed to constitute a significant contribution to the holistic therapeutic benefits of CMs. A presystemic PK–PD focusing on gut drug exposure and gut-originated targets should be included into a global PK–PD strategy to complement the current PK–PD strategies to provide a comprehensive mechanistic understanding of the multi-component multi-target holistic clinical outcomes of CMs.

## Abbreviations

PK: pharmacokinetics
PD: pharmacodynamics
CMs: Chinese medicines
TCM: traditional Chinese medicine
AUC: area under concentration–time curve
ADME: absorption, distribution, metabolism and excretion
P-gp: *P*-glycoprotein
HDI: herb-drug interactions
NMEs: new molecular entities
PBPK: physiologically based pharmacokinetics
